# Application and kinetic evaluation of upflow anaerobic biofilm reactor for nitrogen removal from wastewater by Anammox process

**DOI:** 10.1186/1735-2746-10-20

**Published:** 2013-02-18

**Authors:** Ali Akbar Babaei, Roza Azadi, Nemat Jaafarzadeh, Nadali Alavi

**Affiliations:** 1 Environmental Technologies Research Center, Ahvaz Jundishapur University of Medical Sciences, Ahvaz, Iran; 2 Department of Environmental Health Engineering, School of Public Health, Ahvaz, Jundishapur University of Medical Sciences, Ahvaz, Iran

**Keywords:** Anammox, Nitrogen removal, Kinetics, Stover-Kincannon model, Grau model

## Abstract

The lab-scale upflow anaerobic biofilm reactor was successfully operated for the treatment of synthetic wastewater with high nitrogen load by Anammox (anaerobic ammonium oxidation) process. During the entire period of operation, the reactor temperature was kept at 35±1°C. The operational strategy consisted of both increasing the ammonium and nitrite concentrations from 60 to 700mgN/L and from 80 to 920 mgN/L, respectively and decreasing the hydraulic retention time from 24 to 6 h, at each step. The highest achieved removal efficiency of ammonium and nitrite were 91 and 93%, respectively. Consequently, due to their acceptable performance for nitrogen removal in previous researches, modified Stover-Kincannon and Grau second-order models were used in this study. According to the experiment results, the model validity testing showed that the Stover-Kincannon model was a little more appropriate for the description of nitrogen removal in the reactor, even though both models gave high correlation coefficients (R^2^=0.999).

## Introduction

Due to lower operation cost and energy saving, the use of biological treatment process is recommended in order to treat the effluents with high concentrations of ammonium before discharging them into water resources. A number of biological treatment processes have been used for ammonium removal including: shortcut nitrification-denitrification ammonium removal over nitrite (SHARON), oxygen-limited autotrophic nitrification-denitrification (OLAND), complete autotrophic nitrogen removal overnight (CANON), and anaerobic ammonium oxidation (ANAMMOX) [1,2]. Among the above mentioned processes, Anammox is an innovative process that was discovered in the 1990s and has shown noticeable results for the removal of ammonium and nitrogenous compounds from wastewater [3]. In this process, ammonia in the presence of nitrite as the electron acceptor oxidizes to nitrogen gas under anoxic conditions [4].

Anammox is an energy saving biological nitrogen removal process which was limited to slow growth rate of anammox bacteria during start-up period [5]. It is an autotrophic process that consumes less than 40% oxygen without requiring an organic carbon source for denitrification. It also oxidizes 55-60% of ammonium to nitrite and the remaining of ammonium will be oxidized with nitrite at a reasonable time during this process [6].

The application of Anammox process for ammonia removal has been developed for wastewater treatment of many different reactors, such as landfill leachate in a continuous reactor [7], nitrous organic wastewater in anaerobic sludge blanket reactors (ASBR) [8], anaerobic digester supernatant in sequencing batch reactor (SBR) [9], and supernatant with high concentrations of ammonium in moving bed bioreactor (MBBR) [10]. But few studies have been done to evaluate and determine the substrate removal kinetics of Anammox process [11]. Only three studies were found to evaluate the different kinetic models by describing the nitrogen removal by Anammox process, which was carried out in an Anammox non-woven membrane reactor [12], in an Anammox upflow filter [11] and in an Anammox upflow bioreactor [13].

Process modeling can be applied to control and evaluate the performance of the treatment plant, as well as optimizing the plant design and scaling up the pilot plant investigations [14]. Although different kinetic models were used to study the substrate removal in anaerobic biological process, modified Stover-Kincannon and Grau second order models seem to be the best for describing nitrogen removal [11-13].

The purpose of this research was investigating the application of Anammox process in the upflow anaerobic biofilm reactor for the treatment of synthetic wastewater with high concentrations of ammonium and nitrite. Furthermore, another purpose of this study was determining the kinetics of the Anammox process by the modified Stover-Kincannon and Grau second order models for describing the nitrogen removal in upflow anaerobic biofilm (UABF) reactor.

### Kinetic approach

#### The modified stover-kincannon model

The initial formula of Stover-Kincannon model for the rotating biological contactor (RBC) is [15]:

(1) $$\frac{\mathrm{dS}}{\mathrm{dt}}=\frac{Q\left(S_{0}-S_{e}\right)}{V}=\frac{U_{\max}\left(\frac{QS_{0}}{A}\right)}{K_{B}+\left(\frac{QS_{0}}{A}\right)}$$

Where dS/dt is the substrate removal rate (mg/L.d); Q is the flow rate (L/d), V is the reactor liquid volume (L), S_0_ and S_e_ are the influent and effluent substrate concentrations (mg/L), respectively and A expresses the total disc surface area on which there is immobilized biomass concentration. U_max_ represents the maximum removal rate of substrate (g/L.d) and K_B_ is the constant of saturation value (g/L.d). In this model, the suspended biomass concentration is compared with the attached biomass. If instead of the disc surface area (A) we insert the reactor working volume (V), the original Stover-Kincannon model will be modified as follows [16]:

(2) $$\frac{\mathrm{dS}}{\mathrm{dt}}=\frac{Q\left(S_{0}-S_{e}\right)}{V}=\frac{U_{\max}\left(\frac{QS_{0}}{V}\right)}{K_{B}+\left(\frac{QS_{0}}{V}\right)}$$

In equation (2) the shape of linear equation can be illustrated as follows:

(3) $$\frac{V}{Q\left(S_{0}-S_{e}\right)}=\frac{K_{B}}{U_{\max}}\times \frac{V}{QS_{0}}+\frac{1}{U_{\max}}$$

U_max_ and K_B_ can be calculated via intercept and slope of the line, respectively.

### Grau second-order substrate removal model

The formula of Second-order substrate removal model is [17]:

(4) $$\frac{S_{0}\mathrm{HRT}}{S_{0}-S}=a+\mathrm{bHRT}$$

In which HRT is the hydraulic retention time and (S_0_-S)/S_0_ expresses the substrate removal efficiency and is symbolized as E. Therefore, the last equation can be written as:

(5) $$\frac{\mathrm{HRT}}{E}=a+\mathrm{bHRT}$$

## Materials and methods

### UABF reactor operation

**Figure 1:**
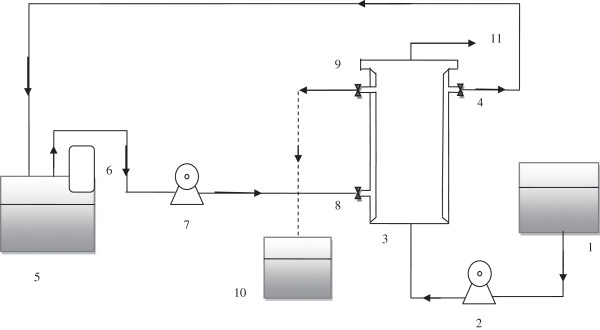
Schematic diagram of the upflow anaerobic biofilm reactor: 1. Feed tank, 2. Peristaltic pump, 3. Reactor, 4. Hot water effluent, 5.Water tank, 6.Thermostat, 7. Recycle pump, 8. Hot water influent, 9. Wastewater effluent and sampling point, 10.effluent collection tank 11. Gas out.

A lab-scale UABF reactor in continuous mode was used for nitrogen removal from synthetic wastewater by the Anammox process (Figure 1). The bioreactor was inoculated with 400 mL of granule sludge. The volatile suspended solid (VSS) of inoculated sludge in the beginning was 12.3 g VSS/L (provided from an upflow anaerobic sludge blanket (UASB) plant, Pegah Milk Factory, Tehran, Iran). The culture of the Anammox sludge was prepared by decreasing the COD/N ratio in the influent gradually and lasted approximately 120 days [18]. The lab-scale bioreactor with the effective volume of 1.8 L consisted of a doubled wall plexiglass cylindrical column (25 cm high), which was comprised of: an inner cylinder (internal diameter of 11 cm and external diameter of 12 cm), the outer cylinder as water jacket (internal diameter of 14 cm and external diameter of 15 cm). Temperature was kept at 35±1°C by a set including a recycling pump used for pumping the circulated water from the water tank to the outer cylinder (according to Figure 1), and a thermostat was installed on the water tank. The body of the reactor was covered with a dark cover to prevent light penetration and algal growth. The plastic media (bee-cell 2000) was filled to 50% of the total reactor volume. This type of media was used as a biofilm support material due to its large surface area (650 m^2^/m^3^) and high porosity (pore volumes up to 87%).

Initially, the bioreactor was operated in a continuous mode with the synthetic wastewater flow rate of 1.8 L/d. pH was kept at a range of 7.5-8.0 by adding sodium bicarbonate. The initial ammonium and nitrite concentrations were 60 and 80 mgN/L, respectively. The operational trend consisted of increasing the concentration of influent nitrogen and decreasing the HRT stepwisely.

### Synthetic wastewater

The composition of the synthetic wastewater that was used for lab-scale upflow bioreactor was (g/L) [19]: NaHCO_3_, 1.25; KH_2_PO_4_, 0.027; CaCl_2_.2H_2_O, 0.3; MgSO_4_.7H_2_O, 0.3. The ammonium and nitrite in the form of (NH_4_)_2_SO_4_ and NaNO_2_ were used as the main influent substrates. 1 mL/L of any trace element solution was added to the above composition. Concentration of the trace elements in each of the solution was as following (g/L):

Trace element NO. 1: EDTA 5.0, FeSO_4_ 5.0.

Trace element NO. 2: EDTA 15.0, ZnSO_4_.7H_2_O 0.43, CoCl_2_ .6H_2_O 0.24, MnCl_2_ .4H_2_O 0.99, CuSO_4_ 5H_2_O 0.25, NaMoO_4_.2H_2_O 0.22, NiCl_2_.2H_2_O 0.19, Na.SeO_4_ .10H_2_O 0.21, H_3_BO_4_ 0.014, and NaWO_4_.2H_2_O 0.050.

### Analysis

The ammonium, nitrite and nitrate concentrations were measured according to standard methods [20]. Ammonium and nitrite were analyzed by the colorimetric method and nitrate was measured by spectrophotometric method. The pH and dissolved oxygen (DO) were measured in obtained samples by a portable pH meter (Metrohm, model 826) and a portable DO meter (Eutech, model 1500), respectively. The biomass of the ammonium oxidizing culture was measured according to standard methods [20]. The soluble COD was measured through colorimetric method by closed reflux method. Each examination was run in triplicate.

## Results

### Process performance

**Figure 2:**
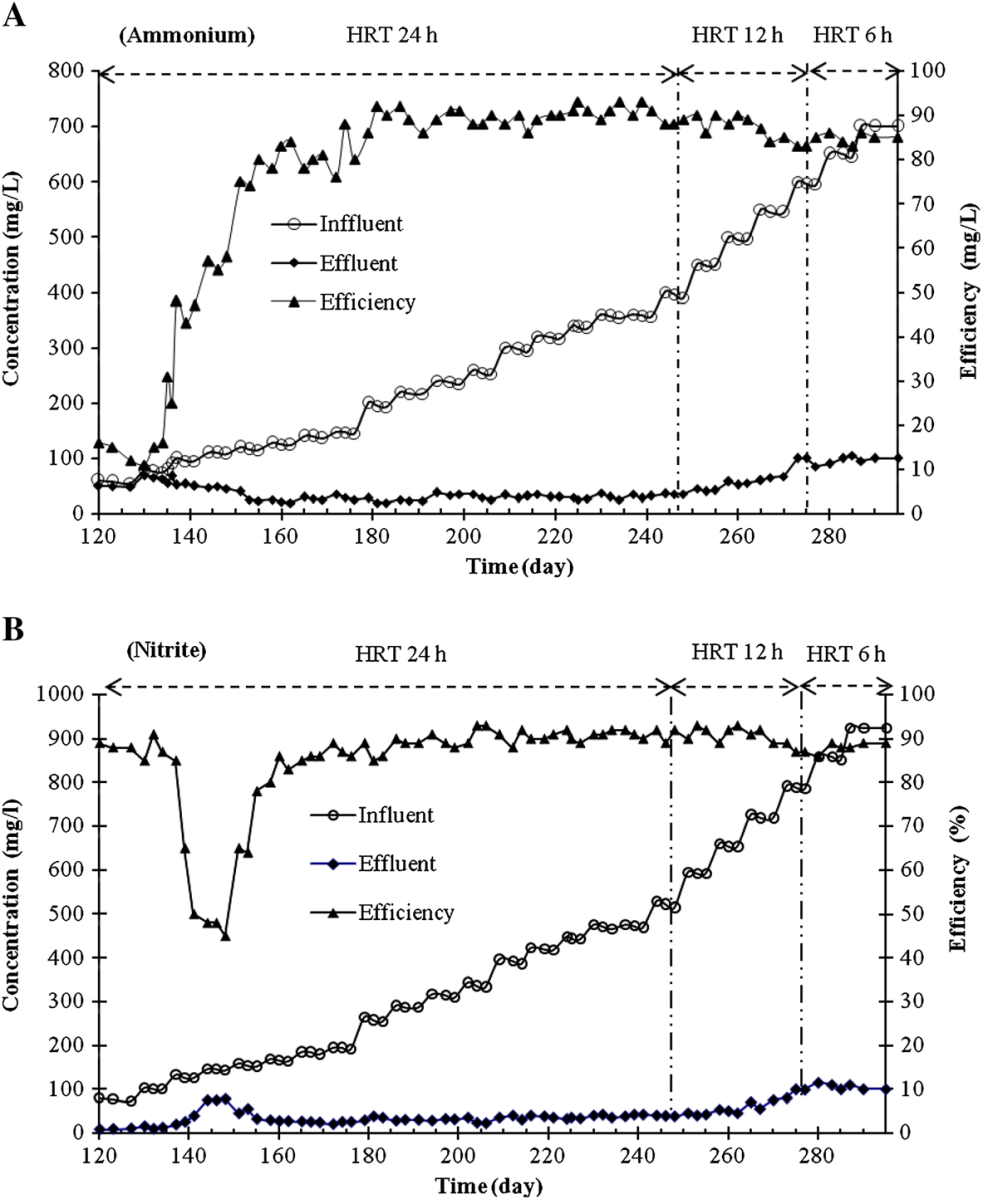
Anammox process perforrmance in UABF reactor during the operation period: (A) Ammonium; (B) Nitrite.

As illustrated in Figure 2 (A, B), the initial 8 weeks of the first operation period can be considered as the acclimatization period (from 120 to 175 days) for the microbial population. During this period of the experiment, due to variation in sludge culture medium, the ammonium removal efficiency was between 11% and 75%, but nitrite removal efficiency (from 117 to 135 day) was between 85% and 91%. However, from day 140 to 160, the biomass encountered a shock of loading rate, and the nitrite removal efficiency decreased from 85% to 45%. The removal efficiency of nitrite and ammonium improved from day 160 and 180, respectively. During the operation period, the influent ammonium and nitrite concentrations gradually increased from 60 to 700 mgN/L and 80 to 920 mgN/L, respectively. Consequently, HRT decreased from 24 to 6 h. Due to the gradual decrease of HRT and increase of nitrogen loading rate to the highest 5 gN/L.d, ammonium removal efficiency reduced from 93% (day 181) to 86% (day 290). During the entire run time, the maximum ammonium and nitrite removal efficiencies were achieved at 91% and 93%, respectively.

The nitrogen conversion rate, determined as the sum of ammonium and nitrite removal rate, was obtained in the range between 0.762 and 5.64 gN/L.d, respectively.

### Calculation of nitrogen removal rate by anammox bacteria

Nitrogen removal rate (NRR) by Anammox bacteria in the UABF reactor was determined based on the stoichiometry reaction of the Anammox process found in the equation below:

(6) $$\mathrm{NRR}=\frac{\mathrm{\Delta N}}{\mathrm{HRT}}$$

$$\begin{array}{l} \mathrm{\Delta N}=\left(\mathrm{Influent}\;\mathrm{ammonium}–\mathrm{effluent}\;\mathrm{ammonium}\right)\\ \;+\left(\mathrm{effluent}\;\mathrm{nitrite}\right)+\left(\mathrm{effluent}\;\mathrm{nitrate}\right) \end{array}$$

**Figure 3:**
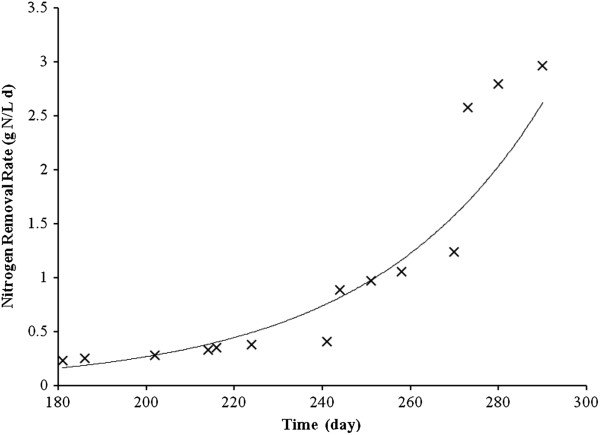
Variations of calculated nitrogen removal rate by Anammox bacteria during the operation period.

Concentrations of influent and effluent ammonium, nitrite and nitrate were at mgN/L, respectively. As illustrated in Figure 3, the nitrogen removal rate increased exponentially from 0.232 to 2.964 gN/L.d, while the nitrogen loading rate (NLR) increased from 0.195 to 2.8 gN/L.d for a long time.

### Modified stover-kincannon model

**Figure 4:**
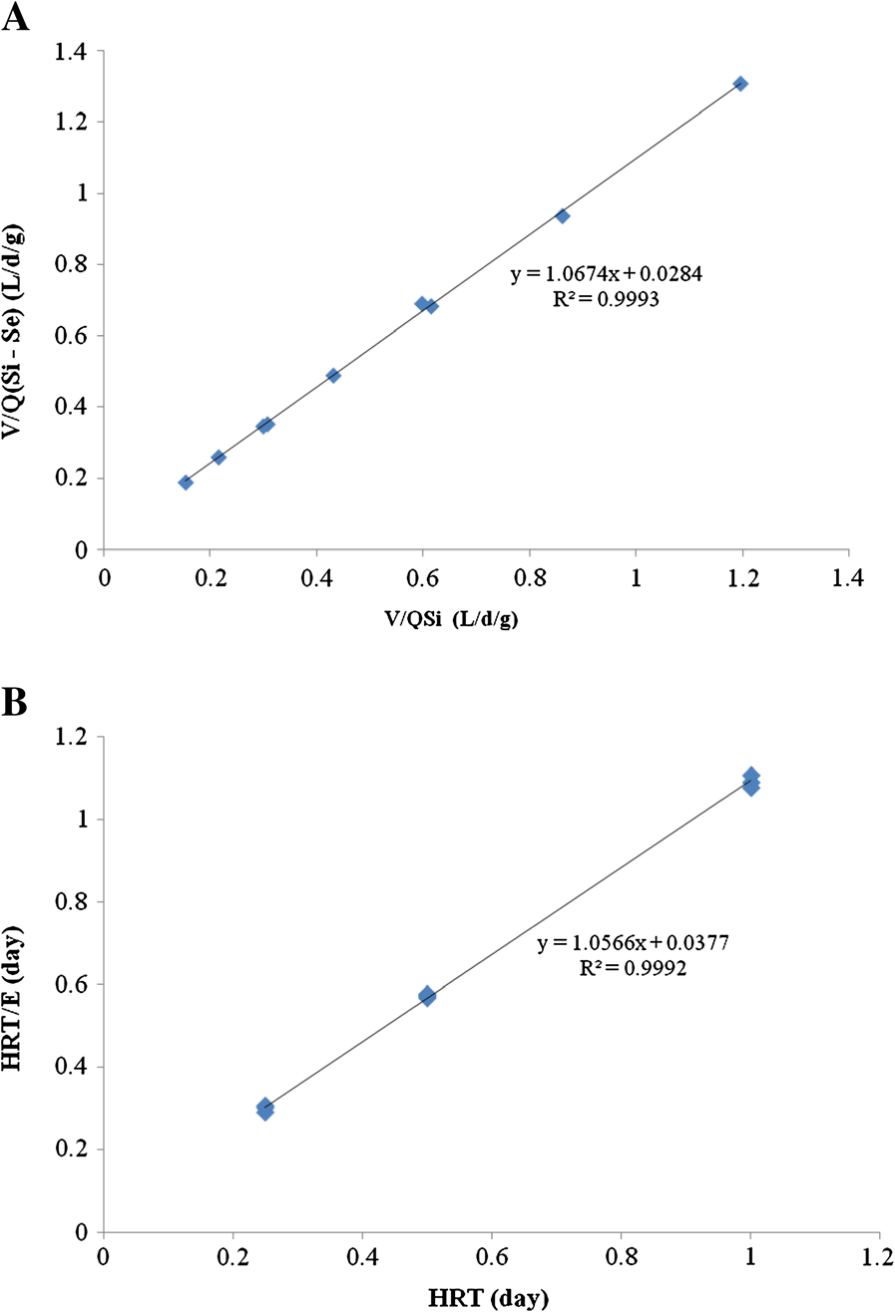
Substrate removal model plot for nitrogen removal in the UABF reactor: (A) The modified Stover-Kincannon model; (B) The Grau second-order model.

Figure 4A shows the kinetic coefficients of the modified Stover-Kincannon model obtained from the plot of V/[Q(S_i_-S_e_)], inverse of the removal rate, versus V/(QS_0_), inverse of the total loading rate. Values of the saturation value constant and the maximum substrate removal rate were determined as 38.107 kg/m^3^.d and 35.71 kg/m^3^.d from the slope and intercept of Figure 4A, respectively. The correlation coefficient was 0.9993, upholding the capability of the modified Stover-Kincannon model.

### Grau second-order substrate removal model

In order to obtain the kinetic coefficients, Equation (5) was plotted in Figure 4B. The value of coefficients (a) and (b) were calculated as 0.037 and 1.056 according to the intercept and slope of the straight line on the graph, respectively. The correlation coefficient of the second-order model was 0.9992. The formula for predicting effluent nitrogen concentrations for the UABF reactor is given by:

(7) $$S=S_{0}\left(1-\frac{1}{1.056+\frac{0.037}{\mathrm{HRT}}}\right)$$

## Discussion

The Anammox process performance in the upflow anaerobic biofilm reactor was evaluated at different NLRs and HRTs using synthetic wastewater with high loading rates. Consequently, kinetic parameter analyses of the reactor were carried out according to the experimental data to determine the most appropriate model in describing the nitrogen removal in the UABF reactor.

As illustrated in Figure 2 (A, B), during the acclimatization period (from 120 to 175 days), predominant bacteria (for example nitrifying bacteria) may be killed due to the conversion of organic nitrogen to ammonium [21], but after 60 days the bacteria were adapted with their environment; therefore, the ammonium and nitrite removal efficiency improved. These results agreed with the results reported by Yang *et al*. (2009) [2].

During the operation period, due to the gradual decreasing of HRT and increase of nitrogen loading rate to the highest value, 5 g N/L d, ammonium removal efficiency reduced from 93% (day 181) to 86% (day 290). While, in the similar study conducted by Cho *et al.* (2010) [22] in anaerobic upflow Anammox reactor, after 8 months of operation, stable total nitrogen removal 60% was reported with periodical removal of excess Anammox granules. From day 270 to 295 (in the higher ammonium concentrations), the Anammox bacteria could not reduce ammonia and nitrite concentrations due to the increasing of nitrogen loading rate, which could probably be toxic to ammonium oxidizing bacteria under anoxic conditions. As a result, ammonium and nitrite concentrations increased in the effluent. The results are strongly similar to the findings of Lopez *et al*. (2008) [23] and Ni *et al*. (2010) [12]. Also, when nitrate was observed in the effluent, the ammonium and nitrite concentrations did not increase significantly.

The nitrogen conversion rate, determined as the sum of ammonium and nitrite removal rate, was obtained in the range between 0.762 and 5.64 gN/L.d, respectively.

As illustrated in Figure 3, the NRR increased exponentially from 0.232 to 2.964 gN/L.d while NLR increased from 0.195 to 2.8 g N/L.d for a long time. Also, Cho *et al.* (2010) reported that the maximum total nitrogen removal rate of 17.4 kgN/m^3^.d was attained at the HRT of 0.43 h on day 259 [22].

The color of sludge changed during the entire process period. The color of sludge before cultivation was a dark color. After ammonium and nitrite removal in the UABF reactor, at the initial concentration of ammonium 60–210 mg N/L, the biomass was grizzle or ashen in color. Also, in high concentrations of ammonium, at approximately 250 mg/L, a maize color, orange or light red, was created which is similar to the Anammox cultures expounded in previous studies [24-26]. However, the color of sludge was fixed at the highest concentration of 400 mg/L, which indicate the application of the Anammox in high concentrations. So, the color change is considered as one of the characteristics of Anammox sludge. In addition, changes in the amount of sludge granules by measuring VSS (in first and end of the operation period), were from 12.3 to 23.8 mg/L. Also observing N_2_ bubbles produced under anaerobic condition is another reason to approve Anammox process.

### Evaluation of the process performance in UABF reactor by the kinetic models

**Figure 5:**
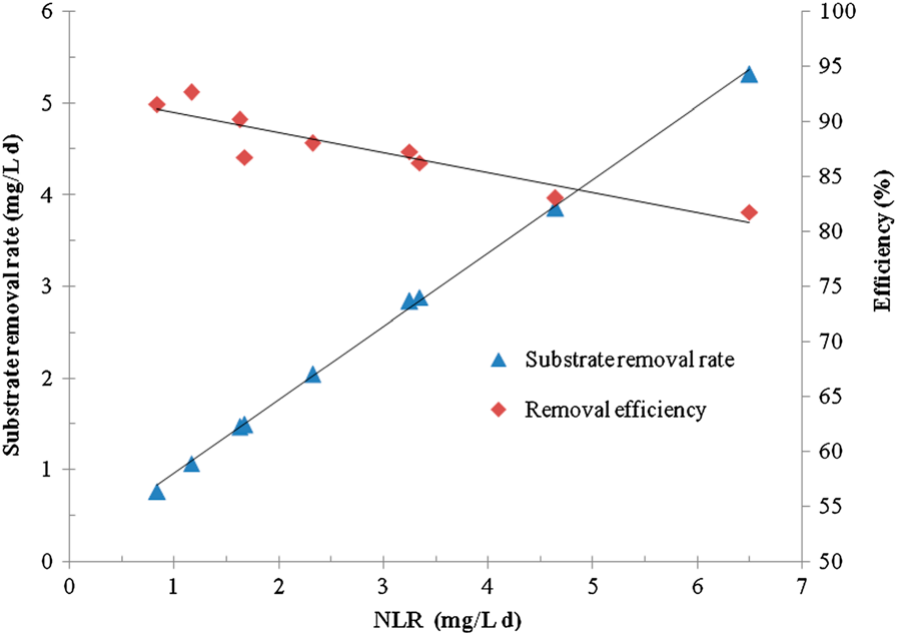
Effect of Nitrogen Loading Rate on Substrate removal rate.

The modified Stover-Kincannon model and Grau second-order model were used to evaluate the Anammox process performance in the UABF reactor. During the kinetic study, the HRT was adjusted at 24 h and gradually reduced to 6 h, whereas the flow rate increased from 1.8 L/d to 7.2 L/d. Also, the influent ammonium and nitrite concentrations were increased from 350 and 470 mg N/L to 700 and 924 mg N/L, respectively. Consequently, The NLR increased gradually from 0.835 gN/L.d to 6.496 gN/L.d. The results obtained from experiments during the kinetic study, indicated a meaningful relationship between the removal efficiency and the hydraulic retention time. As HRT decreased, total nitrogen concentration in the effluent increased from 0.07 g/L to 0.2 g/L. Also, the removal efficiency decreased stepwise from 91.6% to 81.8%. The effect of the nitrogen loading rate on the substrate removal rate is depicted in Figure 5. According to Figure 5, with the increase of nitrogen loading rate, the substrate removal rate increased from 0.765 to 5.316 kg/m^3^d and the nitrogen removal efficiency decreased to 81.8%.

**Figure 6:**
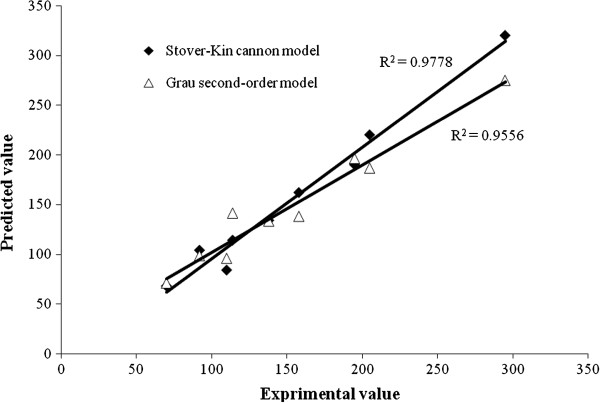
The validity of the modified Stover-Kincannon and Grau second-order models for nitrogen removal.

Figure 6 illustrates the experimental data obtained in this study *vs.* the predicted values for the effluent total nitrogen obtained by the modified Stover-Kincannon and the Grau second-order models in UABF reactor, which were calculated by Equation (7), respectively. It can be observed that the predicted data is in agreement with the experimental data by the modified Stover-Kincannon model (R^2^=0.978) and the Grau second-order model (R^2^=0.956) however the Stover-Kincannon model was more appropriate for nitrogen removal kinetics in UABF reactor. Table 1 compares the constants obtained from the modified Stover-Kincannon and Grau second-order models in the previous studies with coefficients determined in this research. In the current study, *K*_*B*_ and *U*_*max*_ values are larger than those calculated by Jin and Zheng (2009) [11], Ni *et al.*, (2010) [12] and Alavi *et al.*, (2011) [13]. Presumably, this variation in value depends on influent wastewater composition, the type of activated culture applied to the reactor and the type of reactor. The authors believe this indicates that the UABF reactor has a higher capability for the treatment of wastewaters with high nitrogen content.

**Table 1:** Comparison of the kinetic constants in different Anammox reactors

**Reference**	**Grau second-order model**		**Stover-Kincannon model**			**HRT (h)**	**Influent nitrite (mg/L)**	**Influent ammonium (mg/L)**	**Reactor type**	**Substrate**
	**a**	**b**	**K**_**B**_	**U**_**max**_	**R**^**2**^					
Jin and Zheng, [11]	1.397	0.964	12.0	12.4	0.979	10.1-1.99	288-304	270-305	AUF	*
Ni *et al*. [12]	0.105	1.110	8.9	7.89	0.998	69-14.4	153-313	115-297	ANMR	*
Alavi *et al*. [13]	0.052	1.039	21.6	20.7	0.998	24-6	528	400	UABF	**
Present study	0.037	1.056	38.1	35.7	0.999	24-6	475-924	360-700	UABF	***

* Synthetic medium.

** Petrochemical wastewater.

*** High-strength synthetic.

## Conclusions

The anammox upflow anaerobic biofilm (UABF) reactor is an efficient process for nitrogen removal from wastewater. Gradual decreasing of HRT and increasing of NLR reduced nitrogen removal efficiency. The NRR increased exponentially while NLR increased during operation time. The Grau second-order model and the modified Stover-Kincannon model were appropriate to describe the nitrogen removal of the UABF reactor. Even though the correlation coefficient of the both models were high (R^2^=0.999), model testing indicated that the modified Stover-Kincannon model was slightly more suitable for nitrogen removal kinetics in the UABF reactor.

## Competing interests

The authors declare that they have no competing interests.

## Author’s contributions

AAB designed experiments, gave technical support and conceptual advice and drafted the parts of the manuscript. RA, designed and performed experiments, analyzed data. NJ carried out the data processing. NA conceived the strategies, developed the concept, supervised the study and finalized the manuscript. All authors read and approved the final manuscript.
